# Combining traditional anatomy lectures with e-learning activities: how do students perceive their learning experience?

**DOI:** 10.5116/ijme.56b5.0369

**Published:** 2016-02-21

**Authors:** Lukas Lochner, Heike Wieser, Simone Waldboth, Maria Mischo-Kelling

**Affiliations:** 1 Teaching Support Office, Claudiana, College of Health Care Professions, Bolzano/Bozen, Italy; 2 Research Unit, Claudiana, College of Health Care Professions, Bolzano/Bozen, Italy; 3 Library Services, Claudiana, College of Health-Care Professions, Bolzano/Bozen, Italy

**Keywords:** Didactic lecture, anatomy, blended learning, flipped classroom, qualitative study

## Abstract

**Objectives:**

The purpose of this study was to investigate how students perceived their learning experience when combining traditional anatomy lectures with preparatory e-learning activities that consisted of fill-in-the-blank assignments, videos, and multiple-choice quizzes.

**Methods:**

A qualitative study was conducted to explore changes in study behaviour and perception of learning. Three group interviews with students were conducted and thematically analysed.

**Results:**

Data was categorized into four themes: 1. Approaching the course material, 2. Understanding the material, 3. Consolidating the material, and 4. Perceived learning outcome.  Students appreciated the clear structure of the course, and reported that online activities encouraged them towards a first engagement with the material. They felt that they were more active during in-class sessions, described self-study before the end-of-term exam as easier, and believed that contents would remain in their memories for a longer time.

**Conclusions:**

By adjusting already existing resources, lectures can be combined fairly easily and cost-effectively with preparatory e-learning activities. The creation of online components promote well-structured courses, can help minimize ‘student passivity’ as a characteristic element of lectures, and can support students in distributing their studies throughout the term, thus suggesting enhanced learning. Further research work should be designed to confirm the afore-mentioned findings through objective measurements of student learning outcomes.

## Introduction

The traditional didactic lecture is one of the most widely criticized educational methods.^1^^-^^3^ Although it is considered to be efficient for presenting information, providing explanations, and fostering enthusiasm for learning,^4^ it is argued that lectures are particularly ineffective when the instruction goals involve the application of knowledge. Lecture courses usually do not provide enough contact time for deeper learning activities. This is particularly the case if students become passive recipients of large amounts of information, leaving them with limited mental capacity to actively engage in the learning process. As a result, students can postpone their study time (commonly referred to as “cramming before the exam”). Previous faculty development initiatives have therefore focused on increasing interaction with students during lectures as a way to enhance learning.^5^^-^^8^ Nonetheless, criticism of the didactic lecture has not waned, and despite the move away from teacher-centered methods towards more learner-centered curricula, constraints such as large student enrolment and diminishing resources are favouring a return to the traditional lecture in educational settings.^9^

With new technology, the traditional approach to the didactic lecture can be redefined by combining it with online learning. Online learning has been found to be effective, especially when linked to face-to-face instruction in a blended learning format.^10^^, ^^11^ One of the applications of blended learning is the pedagogical model of the Flipped Classroom, in which students engage with some course material prior to a class session.^12^^-^^14^ Teachers can then use classroom time more effectively, e.g. by guiding students through a process of problem solving. However, there is limited research in how students in university education react to this kind of flipping the traditional setting.^15^ It has been criticized that too much emphasis has been put on the possibilities of technology and not enough on the needs of the learners,^16^ and attention has been drawn to the disparity between what the students actually do when studying on-line and what the teachers might imagine, wish or think they are doing.^17^ There is also the concern that online learning might even be detrimental to learning if it is driven by technology rather than the learning process.^18^ In summary, the literature suggests that low-cost and low-tech, but instructionally-sound online learning represents a feasible strategy to follow.^19^ The question to be addressed is, therefore, how can online components be integrated effectively into existing approaches to teaching and learning within established curricula.^20^^-^^24^ Without insight into this issue, we cannot understand the best way to implement educational technology to enhance student learning.

This descriptive study addresses an important issue in medical education, namely, the enhancement of the widely criticized lecture style of teaching by employing pre-lecture preparations which are delivered online. We report on how we enhanced a series of anatomy lectures through e-learning activities and qualitatively investigated students’ reported changes in their studying behaviour and the perception of their learning outcome.

## Methods

### Educational setting

The College of Health-Care Professions in Bolzano/Bozen, Italy, offers three-year bachelor programs in various allied health professions. With the exception of nursing, classes are normally attended by groups of around 20 students. In the discipline-based curricula, the predominant teaching method during theoretical instruction is mandatory attendance in traditional didactic lectures. Assessment consists mainly of written or oral end-of-term exams. This study refers to the first author’s general anatomy lectures of internal organs for the bachelor programs in speech therapy and biomedical laboratory techniques.

### Combing traditional lectures with e-learning activities

The first author (LL) redesigned a 30-hour internal anatomy lecture series into a blended learning format of six sequential organ-based modules. Each module was enhanced through the creation of preparatory online learning activities. In total, seven hours were allocated to online activities, while 23 hours remained as face-to-face lecture time. Therefore, approximately 25% of the original lecture time was allotted to e-learning activities while 75% remained mandatory in-class instruction, ensuring that the overall student workload would remain unchanged. The course was then delivered in the academic year 2013/14 to two groups of students: a class of 20 first-year speech-therapy students and a class of 16 first-year students of biomedical laboratory techniques. The students receive no further instruction in internal anatomy (lectures, tutorials or dissections) elsewhere in the curricula.

The online activities were created and delivered using ILIAS® software. ILIAS® is an acronym that translates into Integrated Learning, Information and Work Cooperation System; it is a German open source web-based learning management system developed at the University of Cologne and published under the GNU General Public License. The first author participated in an 18-hour training course to familiarize himself with the features and operation of the software.

The online learning was conceptualized as six preparatory assignments linked to each of the six modules, and consisted of three sequential steps:

          1. One fill-in-the-blank assignment based on pictures of human anatomy (assignments were created offline from existing lecture material and then uploaded as PDF files. Students were requested to download the material and complete the exercise using a recommended anatomy textbook).

          2. One online human anatomy video and one online video showing a related clinical example (relevant video clips were sourced on the video-sharing website YouTube®, which were then embedded directly into ILIAS®).

          3. An online quiz consisting of 10 multiple-choice questions, eight of which were related to the anatomical images and two to the clinical video (the items were created online using ILIAS®).

To prevent students from working ahead on subsequent modules, we released the online assignments in sequential order immediately prior to their related lecture session. In this way, students had one week to access and complete the online assignments before face-to-face instruction. Students were free to access the online resources on or off-campus. There were no explicit time-slots for completing the online work. The preparatory assignments were not mandatory (students were not required to submit their work, nor was it graded); nonetheless, the assignments were presented as an integral part of the anatomy lecture and students were made aware that the completion and results of the online quiz were electronically tracked and could be checked by the teacher.

The in-class sessions were maintained as didactic lectures and no further activities were added. Anatomy was explained using a “slide and lecture” teaching method in combination with practical demonstrations through medical mannequins and human organ models. Contents also remained the same. However, it was judged that the inclusion of online preparation would reduce the lecture time needed and so the face-to-face contact time was cut by 25%. We ensured that students who did not complete the online preparation work would not benefit inadvertently during contact time; for example, core information of the preparation assignments was reviewed quite quickly, and more focus was directed at students’ questions, understanding and application of anatomical information. In summary, each of the six modules comprised of preparatory online learning (one fill-in-the-blanks assignment, two video clips and one quiz) and one subsequent in-class lecture session, which followed a week after the online activities were released.

### Evaluation methods

A qualitative study was conducted to generate a deeper understanding of students’ study behaviour and their perceived learning outcomes. We selected the group interview format to elicit a variety of opinions in an interactive setting. The study proposal was presented to and approved by the scientific committee of Claudiana, College of Health-Care Professions, Bolzano/Bozen, Italy.

Participant recruitment: All 36 students who had participated in and completed the course were asked to volunteer for group interviews “to evaluate the anatomy lecture”. Fourteen speech-therapy students and seven laboratory-technique students agreed to participate. Three groups were created (two groups of speech-therapy students and one group of students of biomedical laboratory techniques). Participants signed their written informed consent and each participant was assigned a pseudonym.

Data collection: The group interviews were moderated by two authors (HW, SW) and observed by a third author (MMK). None of these individuals was involved in the organisation or teaching of the respective bachelor courses. Prior to the interviews, the first author (LL) familiarized the two moderators with the design and structure of the course, as well as the preparatory online assignments. The interviewers began the group interviews with an open-ended question: “What do think of the anatomy lecture?” which was aimed at examining the participants’ perception of the course.  Further questions included: “Can you describe how you studied for anatomy?”, “In what ways was your learning during the semester and for the exam different from the learning for the exams of your other subjects?”, and in particular, “How did you use the online learning components?” When deemed necessary, the interviewers encouraged the participants to describe their thoughts and perspectives in more detail. The group interviews lasted from 45 to 58 minutes. The interviews were audio-recorded and transcribed verbatim, with identifying information omitted.

Data analysis: We based our analytic procedure on the thematic analysis approach described by Braun and Clarke.^25^ The first author (LL) read the transcripts repeatedly to familiarize himself with the data. He then established codes and allocated them to themes. Following this, he discussed and reviewed the definition and context of the themes with the other authors who were present during the interviews (HW, SW, MMK). To member-check the analysis, we presented the results orally to a volunteer group of four speech-therapy students who had taken part in the group interview; these students fully agreed with the analysis.

## Results

The data was categorized into three sequential themes: 1) Approaching the course material, 2) Understanding the course material, and 3) Consolidating the course material. These themes contributed to a further, fourth theme: Perceived learning outcome. The following description is a summary of what the participants expressed during the group interviews.

### Approaching the course material

The first theme relates to the preparatory online assignments. Some participants stated that these tasks created—in the words of one student—a sort of “positive pressure”. Even though they were not obliged to complete the assignments, they did not want to arrive unprepared for the class and feel “left behind”. This encouraged them towards an early engagement with the material. One student called this “structured freedom”. “Structured” in the sense that the online activities were accurately defined, and “freedom” because they were able to choose for themselves the place and the time of the day to complete them. For one student the assignments were too structured, making him feel cramped, however, he stated that otherwise he probably wouldn’t have started to look at the material as early as he did. Nearly all participants appreciated the video clips because the mental images they created helped them to visualise abstract theoretical concepts. They found the videos useful to obtain a “basic understanding and overview” of the material, but considered them less appropriate for detailed learning. They believed that video clips should not exceed ten minutes. Most participants stated that they liked doing the online quizzes as a way of self-testing, with some participants considering them to be a “challenging game”. They reported that the quizzes “made them think”, but were irritated by questions that referred to the video with the clinical example, since they considered that this video was not relevant to the end-of-term anatomy exam. Some participants felt that online quizzes shouldn’t be completed before the in-class lecture and suggested that it would be better to do them afterwards with more content knowledge to genuinely test themselves. In general, most participants described that the preparatory online work “generated curiosity and questions” leading up to the in-class session; some students reported searching for additional sources of information to those already provided in the virtual learning environment. Most participants stated that they appreciated the possibility of reviewing the online content when it seemed necessary or before the exam; however, they were against devoting more time to online learning at the expense of face-to-face instruction. One female student of the biomedical laboratory techniques program was not in favor of the online learning activities at all and preferred to study from the anatomy textbook only.

### Understanding the course material

The second theme refers to the in-class lecture sessions that followed the online learning. Most participants felt that having a background in the material allowed for “optimal use of the lecture time” with the teacher. They stated that the preparatory assignments “facilitated student involvement” because they were less busy with note-taking and were “free from the worry of missing something important”. They also believed that the background knowledge acquired through online preparation helped them better understand the clinical aspects of the lecture during face-to-face instruction. Most students felt that having foreknowledge of the material gave them a better chance to respond accurately to the teacher’s questions. They reported “a lot of ‘Aha!’ moments” during lecture sessions and they felt that by the end of the class they had fully understood the contents because it had been “explained again from a different perspective”. In fact, some participants described the face-to-face sessions as a “sort of a first revision” of the contents and deemed the in-class lecture “already as study time”. They stated that in other lectures (held in a traditional format) they might also believe at first that they had absorbed the material because “things seemed logical at the time”, but realised subsequently they “knew things only superficially” when they began to review their notes at home in preparation for the end-of-term exam.

### Consolidating the course material

The third theme relates to self-study before the exam. Nearly all participants stated that their self-study was “easier and faster” when compared with other course lectures (held in the traditional format) during the term. They felt that they didn’t have “to start from zero”: firstly, as they had already been in contact with the material on two separate occasions (during the online preparatory activities and the following lecture sessions); secondly, as they felt they had fully understood the contents; and thirdly, as they had prepared thorough and well-structured notes. As a result, most participants stated that they could begin self-study “efficiently on one’s own” and without delay. By contrast, some students reported that when beginning to study for exams of other courses (held in the traditional format) they often needed to contact peers, to photocopy material, or to look things up again, resulting in a loss of precious study-time. For this course, most of these preparations for self-study had already taken place. They also stated that, due to the clear structure of the course, they were confident that they would “study the right things” (i.e., the relevant contents) during their revision for the end-of-term anatomy exam.

### Perceived learning outcome

The fourth theme explores the students’ perceived learning outcome. First of all, most participants reported feeling that learning was “much, much easier” due to the “well-organised structure” of the course; the clear structure helped them to accomplish their studies more efficiently. They also stated that they “engaged with the material more deeply” because the same contents were presented in various ways and from different perspectives (through images, videos, and MC questions, in addition to face-to-face communication and medical mannequin and model presentations during the lecture sessions). They stated that, when compared to other course lectures during the term, learning the material “happened with less effort” and that they believed their long-term retention of the material would be improved.

This participant’s comments encapsulated what many students felt about the combined approach:

“The preparatory [online] work was helpful, as it gave me a first insight into the subject matter. It was repeated and filled with details [during the lecture sessions], and it was possible to ask questions that had come up during preparation. I engaged intensively with the subjects and they are still with me today because they were processed over a long time.” (Speech-therapy student, female)

## Discussion

Although the traditional didactic lecture is one of the most widely criticized educational methods, it remains a commonly used instructional approach in health sciences education. One of the main threats to lecturing is seen in the passive role attributed to students. However, new technology enables us to invigorate the traditional lecture approach. We combined traditional anatomy lectures with online learning and evaluated changes in students’ study behaviour and their perceptions of their learning outcome.

One important practical insight we gained is that the process of using technology to design online activities that are compatible with subsequent in-class lectures obliges the teacher to design a course that is well-organised and well-structured. In fact, poor organization is widely seen as one of the most frequent reasons for ineffective teaching.^26^ Most participants in this study convincingly reported that the clearly-laid-out structure of the course considerably facilitated their learning experience. Once again, it became clear, that our students’ major focus at this point in their studies was to pass the end-of-term exam. They especially appreciated the e-learning activities as part of this exam preparation.

From a more theoretical perspective, we believe that our success in pushing students towards a practice of “spaced” learning is the most important finding of this study, since underestimating preparation time for self-study and resultant ‘crammed learning’ before the exams is a frequent problem in our institution’s lecture-based curricula. Cognitive psychology outlines a variety of general principles and strategies that can have a positive influence on learning and memory,^27^ but one of the most thoroughly studied phenomena is the “spacing effect”.^28^^-^^30^ More than a century of research has shown that when a fixed amount of study time is spaced over multiple sessions, retention of long-term knowledge is improved. Most participants in this study highlighted that they had engaged with the material more frequently and over a longer period of time when compared to other traditional didactic lectures. A few participants explicitly stated that they considered the in-class lecture sessions to be a kind of study time. This is of particular interest, as teaching in higher education settings is traditionally seen as a constructional process designed to ensure that students learn outside of the classroom;^31^ our findings suggest that the online preparatory activities can enable students to achieve a significant part of their learning inside the classroom. Considering the intense workload of three-year bachelor programs with mandatory attendance of lectures in health professions education institutions (such as our own), this is a highly relevant issue. If students do not succeed in spreading their learning evenly throughout the term, they will resort to the superficial learning strategy of massed self-study just before the exam.^32^^, ^^33^ Participants in this study often explained how they believed that the online assignments supported them in distributing their learning, suggesting an enhanced learning experience.

We see several limitations in this study, and further educational research is needed to validate the findings across various institutional settings with diverse lecturers and student samples. A potential bias might be that the first author as module developer and lecturer also served in the role of interpreting the interview data. To minimize this possible bias, interpretations were reached upon consensus between all four researches, and the results were member-checked with a group of interview participants. Furthermore, the decision to have the interviews on a voluntary basis lead to a participation of 21 out of 36 students with the potential to introduce a selection bias. Future work should be designed to gain more data regarding the group of students unwilling or unable to attend the interviews. Finally, as we did not measure student learning outcomes objectively in this study, the next logical step would be to compare the performance of students that were in the technology-enhanced group versus those that received a traditional lecture to test whether students’ perceptions of enhanced learning matches demonstrated improvements in (long-term) learning outcomes. This could be especially pertinent if the traditional didactic lecture remains a widely-used instructional method in medical education and if resource constraints increase their use in the future.

This study suggests that it is possible to improve how students view their own learning in institutions of health sciences education where the traditional didactic lecture is the principal educational method. When the basic concept, structure and content of a pre-existing lectures is combined with online learning by adapting existing content material and using freely available video clips, this technology can become a fairly easy-to-implement and cost-effective tool that facilitates the sequential delivery of preparatory assignments, encouraging students to distribute their study over a longer period of time. The students at our institution seemed pleased with the distribution ratio of 25% online learning to 75% face-to-face instruction. Even though they have grown up with online technology, they appreciate the contact time and want it to form the greater part of their learning experience. We used this tool for small groups of students, but it could be applied to much larger groups without increasing preparative work or costs.

## Conclusions

This study addresses an important issue in medical education, namely, the enhancement of the widely criticized lecture style of teaching by engaging students more actively during class through the use of preparatory online learning components. It describes the development, implementation and evaluation of a traditional anatomy lecture series combined with preparatory e-learning activities. By using and adjusting already existing resources these components can be developed and implemented fairly easily and cost-effectively. The results of the evaluation suggest that a didactic lecture can be enhanced through the creation of online learning activities by obliging the teacher to create a well-structured course, by encouraging students towards an early engagement with the course material, and by preventing students from assuming a predominantly passive role during in-class sessions. Students believed that they engaged more actively and deeply with the course material, and reported that they distributed their studies more evenly throughout the term. This led to a reduction of delayed self-study and consequent ‘mass cramming’ before exams. The findings suggest that the students experienced enhanced learning. Future research should be designed to confirm these findings through objective measurement of student learning outcomes.

### Acknowledgements

We would like to thank all the students from the speech-therapy and laboratory-techniques courses who participated in the group interviews, and acknowledge Philip Darbyshire for his comments and suggestions regarding this manuscript.

### Conflict of Interest

The authors declare that they have no conflict of interest.
